# Intensified neoadjuvant radiochemotherapy for rectal cancer enhances surgical complications

**DOI:** 10.1186/1471-2482-13-43

**Published:** 2013-09-30

**Authors:** Leif Schiffmann, Nicole Wedermann, Michael Gock, Friedrich Prall, Gunther Klautke, Rainer Fietkau, Bettina Rau, Ernst Klar

**Affiliations:** 1 Department of General, Thoracic, Vascular and Transplantation Surgery, University of Rostock, Schillingallee 35, Rostock 18057, Germany; 2 Institute of Pathology, University of Rostock, Strempelstr. 14, Rostock 18055, Germany; 3 Department of Radiotherapy, Sozialstiftung Bamberg, Buger Str. 80, Bamberg 96049, Germany; 4 Department of Radiotherapy, University of Erlangen, Universitätsstr. 27, Erlangen 91054, Germany

**Keywords:** Rectal cancer, Intensified neoadjuvant radiochemotherapy, Postoperative complications, Anastomotic leakage

## Abstract

**Background:**

Neoadjuvant radiochemotherapy has proven superior to adjuvant treatment in reducing the rate of local recurrence without impairing cancer related survival or the incidence of distant metastases. The present study aimed at addressing the effects of an intensified protocol of neoadjuvant treatment on the development of postoperative complications.

**Methods:**

A total of 387 patients underwent oncological resection for rectal cancer in our institution between January 2000 and December 2009. 106 patients received an intensified radiochemotherapy. Perioperative morbidity and mortality were analyzed retrospectively with special attention on complication rates after intensified radio-chemotherapy. Therefore, for each patient subjected to neoadjuvant treatment a patient without neoadjuvant treatment was matched in the following order for tumor height, discontinuous resection/exstirpation, T-category of the TNM-system, dividing stoma and UICC stage.

**Results:**

Of all patients operated for rectal cancer, 27.4% received an intensified neoadjuvant treatment. Tumor location in the matched patients were in the lower third (55.2%), middle third (41.0%) and upper third (3.8%) of the rectum. Postoperatively, surgical morbidity was higher after intensified neoadjuvant treatment. In the subgroup with low anterior resection (LAR) the anastomosis leakage rate was higher (26.6% vs. 9.7%) and in the subgroup of patients with rectal exstirpations the perineal wound infection rate was increased (42.2% vs. 18.8%) after intensified radiochemotherapy.

**Conclusions:**

In rectal cancer the decision for an intensified neoadjuvant treatment comes along with an increase of anastomotic leakage and perineal wound infection. Quality of life is often reduced considerably and has to be balanced against the potential benefit of intensifying neoadjuvant radiochemotherapy.

## Background

For advanced rectal cancer, neoadjuvant radiochemotherapy has been proven to reduce the rate of local recurrence in comparison to postoperative treatment [1]. German guidelines state exact treatment rules for UICC stage I to III and localization of cancer in the rectum [2]. The decision for a neoadjuvant treatment is based on local staging. Since there has been no impact of neoadjuvant treatment on cancer related survival or distant metastases [1], effort was taken to improve the systemic results of the neoadjuvant radiochemotherapy (RCT). By adding a second drug to the neoadjuvant radiochemotherapy, the rate of complete responses and tumor regression grade could be increased [3-5]. A complete response has been shown to be a predictive marker for disease free and cancer related survival. Thus, an intensified neoadjuvant RCT protocol was introduced at several institutions including irinotecan or oxaliplatin [5-15].

A potential increase of perioperative morbidity following an intensified radiochemotherapy has not been reported so far [4,5].

The aim of this study was to investigate, whether an intensified neoadjuvant radiochemotherapy leads to an increase of perioperative surgical morbidity.

## Methods

All patients treated for rectal cancer with an oncological resection in our institution between January 2000 and December 2009 were included into this retrospective study after identification by the pathological data base. The term rectum carcinoma was applied to adenocacinomas located at a distance from 0 to 16 cm from the anal verge measured by rectoscopy. The cancer was located in either the lower (0- < 6 cm), middle (6- < 12 cm) or upper (12-16 cm) rectum. Patients’ records were analyzed under special consideration of neoadjuvant treatment, type of operation and perioperative complications.

According to German guidelines, there was an indication for neoadjuvant RCT for T3, T4 and /or nodal positive tumors of the lower and middle third of the rectum. In the upper third of the rectum, the only indication for neoadjuvant treatment was a T4 cancer. In our facility, the majority of neoadjuvant treated patients received an intensified neoadjuvant radiochemotherapy, which changed within the observation period. From January 2000 to January 2002 patients received a combination of a continuous infusion 5-FU (250 mg/m^2^ per day) over 31 days, seven weekly applications of irinotecan (40 mg/m^2^) and a local radiation five days a week with a single dose of 1.8 Gy adding up to 50.4 Gy (last three doses were reduced). From February 2002 5-FU was substituted by a daily intake of Capecitabine with a single dose between 1000 and 1650 mg/m^2^. Doses of radiation were no longer reduced and reached a cumulative dose of 55.8 Gy. Oxaliplatin had been applied instead of Irinotecan in eight patients.

The type of surgery depended on localization of the tumor, preoperative stool incontinence and general condition of the patient. Generally, patients received a total mesorectal excision (TME) for all cancers located between 0 and 12 cm and a partial mesorectal excision (PME) for all cancers located higher than 12 cm. All anastomoses were performed by double stapling technique. Postoperatively, in case of unusual elevation of CRP or white blood cell count, clinical symptoms as well as suspicious drain secretion, diagnostics were performed to determine an anastomotic leakage by rectal digital examination, water soluble contrast study, endoscopy or ct-scan. If any of the diagnostic tools showed an anastomotic leakage it was documented as such regardless of the clinical consequences (stage I-III).

**Figure 1:**
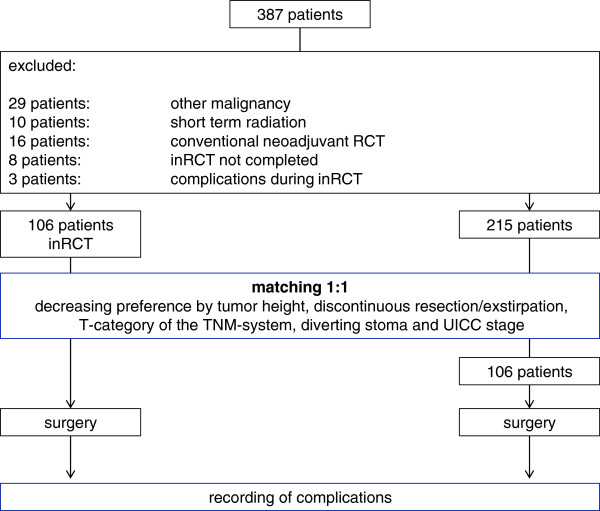
This flow chart shows the matching of the patients and the reasons of exclusions from the study.

After identifying all patients with a rectal adenocarcinoma, we eliminated all patients receiving a short term radiation (5×5 Gy), conventional neoadjuvant radiochemotherapy and all patients having complications during the intensified neoadjuvant treatment (11 patients) or having another malignancy in their history (5 patients). Thereafter, the study population was divided into patients with intensified neoadjuvant RCT and without any neoadjuvant treatment. There were 106 patients eligible to the intensified neoadjuvant RCT group. After that, 106 patients of the not neoadjuvant treated group were matched in decreasing preference by tumor height, discontinuous resection/exstirpation, T-category of the TNM-system, diverting stoma and UICC stage (Figure 1). Of these 106 patients, 27 patients received a combination of 5FU, Irinotecan and 50.4 Gy; 9 patients received a combination of Capecitabine, Irinotecan and 50.4 Gy; 20 patients received a combination of 5FU, Irinotecan and 55.8 Gy; 42 patients received a combination of Capecitabine, Irinotecan and 55.8 Gy; 8 patients received a combination of 5FU, Oxaliplatin and 50.4 Gy.

The study was approved by the Medical Ethical Committee of Rostock University.

### Statistical analysis

Statistical analysis was performed using Statistical Package for Social Science (SPSS) version 15.0. Statistical analysis was done using Pearson’s chi-square test or Fisher’s exact test.

## Results

Between January 2000 and December 2009 387 patients were operated for rectal cancer. In this cohort 106 patients were identified who received an intensified neoadjuvant RCT. These patients were matched with 106 patients, who did not receive any neoadjuvant treatment, for tumor height, discontinuous resection/exstirpation, T-category of the TNM-system, diverting stoma and UICC stage. To rule out, that only the good cases were matched from the not neoadjuvantly treated group, we analyzed the not neoadjuvantly treated group in terms of matched and not matched patients. The main differences were, that the not matched patients had a higher tumor localization and a more frequent operative revision.

Table 1 shows the matching results. As expected, the number of evaluated lymph nodes was higher in patients without neoadjuvant treatment.

**Table 1:** Patient and cancer characteristics for patients with vs. without intensified neoadjuvant RCT

	**With neoadjuvant treatment% (n = 106)**	**Without neoadjuvant treatment% (n = 106)**	**p-value**
**Patients:**	50	50	
Gender ratio (f : m)	1 : 2.92	1 : 1.35	0.01
Age	62.3	68.5	0.067
Comorbidity	68.6	84.6	0.009
Pulmonary	5.7	14.4	0.041
Cardiovascular	21.9	25.0	0.627
Renal	6.7	11.5	0.239
Diabetes	11.4	19.2	0.128
Hypertension	40.0	56.7	0.019
Others	39.0	62.5	0.001
ASA score (mean)	2.28	2.38	0.228
BMI (mean)	26.4	25.9	0.500
Diverting stoma (continous resections only)	76.6	71.0	0.545
Discontinous resection	38.7	40.6	0.888
Rectum exstirpation	29.2	30.2	1.000
Preexisting fecal insuffiency (discontinous resections only)	8.7	17.4	0.665
Infiltration of anal sphincter (discontinous resections only)	54.2	47.8	0.773
Close distance to anal sphincter (discontinous resections only)	43.5	31.8	0.542
Infiltration (pT)			0.026
ypT0	8.5	0.0	
(y)pT1	5.7	10.4	
(y)pT2	27.4	33.0	
(y)pT3	54.7	52.8	
(y)pT4	3.8	3.8	
Lymph node metastasis (pN)			0.945
(y)pN0	52.8	53.3	
(y)pN1	27.4	28.6	
(y)pN2	19.8	18.1	
Number of nodes examined	15.3	18.8	0.001
UICC stage			0.116
UICC 0	3.8	0.0	
UICC I	24.5	32.1	
UICC II	17.0	17.0	
UICC III	31.1	35.8	
UICC IV	23.6	15.1	
Localization			0.986
Upper Rectum	3.8	3.8	
Middle Rectum	40.4	41.5	
Lower rectum	55.8	54.7	
Tumorheight (cm)	5.26	5.31	0.199

Patient characteristics were recorded within three days before surgery, tumor heights was documented at the time of staging and cancer characteristics were taken from the pathological report.

Table 2 shows the complications of the two groups. There were no differences in mortality and overall complications. Non surgical complications were higher in the not neoadjuvant treated group. Surgical complications were significantly different. The anastomosis leakage rate was 3 fold higher in the neoadjuvantly treated group. There was a difference in leakage rate between men and women in the study-group (16.1%) and within the control group (13.5%), which did not reach statistical significance. The perineal wound infection rate in patients with a rectum exstirpation was more than 2-fold, the revision rate was more than 3-fold higher after neoadjuvant RCT. The overall surgical morbidity is also significantly higher after neoadjuvant RCT.

**Table 2:** Postoperative morbidity stratified by intensified neoadjuvant radiochemotherapy

	**With neoadjuvant treatment% (n = 106)**	**Without neoadjuvant treatment% (n = 106)**	**p-value**
30 day mortality	0.0	1.9	0.244
Complications	53.8	50.5	0.550
Non surgical	11.3	23.1	0.028
Urinary infection	2.8	9.6	0.048
Pneumonia	3.8	6.7	0.371
Cardiopulmonary events	0.9	4.8	0.117
Surgical	50.0	31.7	0.018
Wound infections (any grade)	20.8	9.6	0.034
Perineal wound infections (rectum exstirpation only)	42.2	18.8	0.032
Anastomosis leakage (LAR only)	26.6	9.7	0.020
Operative revision (LAR only)	20.3	6.5	0.035

After administrating intensified neoadjuvant treatment, there is no increase in non surgical morbidity, but a severe increase in surgical morbidity like anastomosis leakage or wound infections.

## Discussion

We retrospectively analyzed the postoperative course of patients with rectal cancer subjected to intensified neoadjuvant RCT in comparison with patients who were not treated before surgery. The question was, whether patients treated before surgery had a higher rate of morbidity and mortality compared with the group that did not receive a neoadjuvant treatment. The main finding of this study is that intensified neoadjuvant radiochemotherapy resulted in a significantly higher surgical morbidity rate. To rule out known risk factors, this study was designed as matched pair analysis with matching patients 1 to 1 in decreasing priority for tumor height, discontinuous resection, tumor infiltration, dividing stoma and UICC stage. Patients in the neoadjuvantly treated group were younger and had less comorbidities without affecting the average ASA score. The number of examined lymph nodes was lower after intensified neoadjuvant RCT complying with previous reports [16]. Some patients had a total reduction of the tumor and had therefore an ypT0 and/or UICC 0 classification. In conclusion, matching was successful and the groups were comparable.

Patients without neoadjuvant RCT were older and had a higher rate of comorbidities. This propably was the reason for an increased non-surgical morbidity. The group without neoadjuvant treatment had a leakage rate of 9.7% and was comparable with the results of other institutions [17,18]. But nevertheless the overall surgical morbidity after intensified neoadjuvant treatment (especially anastomotic leakage of 26.6% and perineal wound infection rate of 42.2%) in our series is rather high compared to other groups as shown in Table 3 ranging between 0% and 25.9%. The quality of surgery appears to be comparable with other groups represented by the number of lymph nodes harvested [19]. Other groups reported that there was no increase of surgical morbidity after applying non-intensified neoadjuvant radiochemotherapy (Table 3) [1,20]. By adding an extra agent, surgical results seem to be influenced in a negative way [21]. If there was a benefit in oncologic outcome and if this potential benefit would compensate the increased surgical morbidity, it remains yet uncertain.

**Table 3:** Overview of surgical complications: comparison of neoadjuvant R(C)T with and without intensification to our results

	**Subject**	**30 day mortality (%)**	**Anastomotic leakage (%)**	**Operative revision of anastomotic leakage (%)**	**Perineal wound infection (%)**
Own results (n = 212)	+/- intensified RCT	0 / 1.9	26.6 / 9.7	20.3 / 6.5	42.2 / 18.8
Sauer et al. [1] (n = 823)	Pre/post-operative RCT	0.7 / 1.3	11 / 12		10 / 8^+^
Bosset et al. [20] (n = 1011)	RT/RCT	1.2 / 2.4			
Kapiteijn et al. [19] (n = 1861)	+/- RT	No difference	No difference		26 / 18
Voelter et al. [8] (n = 33)	Intensified RCT	3	6		58
Horisberger et al. [21] (n = 59)	All patients / major/minor response to intensified RCT	3.4	15.5	15.5	
		6.1 / 0	25.9 / 0	25.9 /0	
Gollins et al. [14] (n = 46)	Intensified RCT	0	6.4		22.2^§^
Aschele et al. [22] (n = 747)	Intensified (Oxaliplatin)/"not intensified" RCT	1 / 1	2 / 1		9 / 9&
Sato et al. [23] (n = 67)	S-1 plus Irinotecan	0	0	0	0
Garlipp et al. [24] (n = 2085)	+/- preoperative chemoradio-therapy		12.4 / 12.7	5.5 / 7.5	
Fucini et al. [25] (n = 80)	+/- preoperative RCT	2 / 0	14.8 / 9.1		

^+^delayed healing, ^§^wound dehiscence, &other complication/undetermined.

Taking the literature into account as shown in Table 3, the intensivation of neoadjuvant RCT according to Horisberger et al. [21] appears to be even more aggressive compared to a neoadjuvant treatment without intensivation [1,24] and results in a higher surgical complication rate. On the other hand, Gollins et al. [14], Aschele et al. [22], Sato et al. [23] as well as Voelter et al. [8] report rather low anastomotic leakage rates of 6.4%, 2%, 0% and 6% respectively from a group of 31, 747, 67 and 21 patients with sphincter sparing surgery. All studies concerning an intensified neoadjuvant RCT regime lack either a substantial number of patients and are basically series without a control group or do not focus on the surgical outcome [5,15,22,23]. So far, there has been no explanation, how an additional chemotherapeutic agent could influence surgical morbidity of a subsequent operation which usually takes place 6 weeks after termination of radiochemotherapy. Horisberger et al. [21] found a relationship between tumor response to intensified neoadjuvant therapy and major complications. The rate of anastomotic leakages was 25.9% in the group with a major response comparing to 0% in the group with a minor response to the neoadjuvant treatment. The authors suggest that collagen deposition, the depressing effect on the blood cells and other essential elements of wound healing as well as different definitions of anastomotic dehiscence and the irritation of bowel mucosa could have influenced this result. While the large multicenter studies on the oncological impact of radio(chemo)therapy [1,19,20] do not show differences in the anastomotic leakage rate with or without pre- or postoperative radio-(chemo)-therapy, we demonstrate in our study the results of a single center institution with a standardized and reproducible treatment concept surgically as well as perioperatively. It should be pointed out, that surgical morbidity and mortality was not the main focus of the studies mentioned above whereas surgical complications were the main aim of our investigation. However, in a retrospective multicenter study Garlipp et al. [24] focused on the effect of neoadjuvant radiochemotherapy on the anastomotic leak rate and did not find any differences between groups, even though the tumor location was significantly lower in patients subjected to neoadjuvant treatment. Weiss et al. [5] report pooled data from three trials administering neoadjuvant RCT with capecitabine and oxaliplatin with or without cetuximab. The leakage rate is reported to be 11 per cent of all included patients without stating the fractions of patients with anterior resection or exstirpation of the rectum. Gerard et al. [15] report low anastomotic leakage rates of 6.2% (12 out of 195 patients) administering only capecitabine and 4,9% (10 out of 205 patients) using capecitabine and oxaliplatin in the neoadjuvant RCT regime. Aschele et al. [22] and Sato et al. [23] report extremely low anastomotic leakage rates of 2% and 0%. This is noteworthy since the generally accepted leakage rate after rectal resection reported from leading surgical departments ranges from 5.5% to 37.5% [1,24-32]. Also, a recent study showed a threefold higher anastomotic leakage rate in males in comparison to females after laparoscopic rectal resections [33]. This correlation was somewhat debatable in the past [32]. In our study, there was also a difference between males and females, but due to smaller patient numbers, we did not reach significant differences.

There are only few studies addressing perineal wound infection rate after neoadjuvant treatment in the literature. Data from Gollins et al. [14] as well as Voelter et al. [8] show a higher rate of perineal wound infections after an intensified regime (22.2 and 58%) compared with 10% and 26% after conventional preoperative radiochemotherapy [1,19]. Our study confirms a high incidence of perineal wound infection following intensified neoadjuvant radiochemotherapy.

## Conclusions

In conclusion intensified neoadjuvant radiochemotherapy in rectal cancer patients resulted in a higher surgical complication rate compared with patients without neoadjuvant RCT in our institution as demonstrated by this retrospective matched pair analysis. Our results are in accordance with previous studies in the literature concerning a high rate of perineal wound infections after rectal exstirpation, but not with respect to the demonstrated increase of anastomotic breakdown following resection. However, most of these studies have the draw-back that surgical complications were not the main focus and therefore the key parameters were not analyzed in detail. Further studies are required to substantiate our findings and to investigate whether an increase in surgical complication rate is warranted by a significant improvement of oncological outcome.

## Competing interests

The authors declare that they have no competing interests.

## Authors’ contributions

LS, NW and MG conceived and coordinated the study, collected patients’ data and participated in the statistical analysis. LS drafted the manuscript. GK, FP, RF, BR and EK participated in preparing and drafting the manuscript. All authors read and approved the final manuscript.

## Pre-publication history

The pre-publication history for this paper can be accessed here:

http://www.biomedcentral.com/1471-2482/13/43/prepub
